# Affective Interaction with a Virtual Character Through an fNIRS Brain-Computer Interface

**DOI:** 10.3389/fncom.2016.00070

**Published:** 2016-07-12

**Authors:** Gabor Aranyi, Florian Pecune, Fred Charles, Catherine Pelachaud, Marc Cavazza

**Affiliations:** ^1^School of Computing, Teesside UniversityMiddlesbrough, UK; ^2^CNRS - LTCI, Telecom ParisTechParis, France; ^3^School of Engineering and Digital Arts, University of KentCanterbury, Kent, UK

**Keywords:** brain-computer interfaces, fNIRS, neurofeedback, affective computing, virtual agents

## Abstract

Affective brain-computer interfaces (BCI) harness Neuroscience knowledge to develop affective interaction from first principles. In this article, we explore affective engagement with a virtual agent through Neurofeedback (NF). We report an experiment where subjects engage with a virtual agent by expressing positive attitudes towards her under a NF paradigm. We use for affective input the asymmetric activity in the dorsolateral prefrontal cortex (DL-PFC), which has been previously found to be related to the high-level affective-motivational dimension of approach/avoidance. The magnitude of left-asymmetric DL-PFC activity, measured using functional near infrared spectroscopy (fNIRS) and treated as a proxy for approach, is mapped onto a control mechanism for the virtual agent’s facial expressions, in which action units (AUs) are activated through a neural network. We carried out an experiment with 18 subjects, which demonstrated that subjects are able to successfully engage with the virtual agent by controlling their mental disposition through NF, and that they perceived the agent’s responses as realistic and consistent with their projected mental disposition. This interaction paradigm is particularly relevant in the case of affective BCI as it facilitates the volitional activation of specific areas normally not under conscious control. Overall, our contribution reconciles a model of affect derived from brain metabolic data with an ecologically valid, yet computationally controllable, virtual affective communication environment.

## Introduction and Rationale

One major challenge for brain-computer interfaces (BCI) is to be based on sound computational models grounded in Neuroscience findings. Recent years have seen an interest in developing affective BCI (Mühl et al., 2014) to support a wide range of interactive systems, as an extension of work in affective computing. However, unlike with imagery-based or motor-oriented BCI, the anatomical localization of emotional responses is often elusive (Lindquist et al., 2012), hampering the design of neuroscience-inspired computational models. From a similar perspective, Mattout (2012) has advocated the use of social neuroscience signals to develop improved BCI which would go beyond the traditional command paradigm of conversion of signal into action, and support more sophisticated, life like interactions.

In this article, we report on an experimental framework and investigate affective BCI in a synthetic but realistic situation, in which the user interacts with a virtual agent endowed with facial expressions. Our objective is to study under which conditions users can engage with a virtual agent that displays realistic non-verbal behavior, using only their mental disposition towards the agent. Hence it aims at reconciling some limited but corroborated neuroscience data about mental disposition with what is perhaps the largest body of work in affective computing, facial expressions.

In this framework, we are creating a new vision of *rapport* (Gratch et al., 2007), which we claim to be more controllable than empathy (Light et al., 2009), requiring generally a stronger context and background, or alignment (Menenti et al., 2012) in which verbal communication is required on both sides. We have opted for a Neurofeedback (NF) paradigm, which is appropriate to the closed-loop setting required to investigate rapport. In this system, the input signal is constituted by prefrontal cortex (PFC) asymmetry measured through functional near infrared spectroscopy (fNIRS; capturing the mental disposition towards the agent), and the feedback channel is constituted by the virtual agent’s non-verbal behavior, facial expressions in particular, responding to the perceived disposition. Other supporting elements include the fact that NF facilitates BCI input where the signal is not under direct volitional control, and that PFC asymmetry has been demonstrated to be amenable to NF control (Rosenfeld et al., 1995; Aranyi et al., 2015b).

We have designed an asymmetric situation, in which the user interacts through BCI alone, whilst the virtual agent uses realistic non-verbal behavior. This setting is intended to facilitate the controllability of the experiment, as well as the principled use of background knowledge in affective neuroscience and affective psychology to support the computational approach.

## BCI: Prefrontal Asymmetry and fNIRS

Despite difficulties in identifying neural substrates for major categories of emotions (Lindquist et al., 2012), a large body of work has demonstrated a strong correlation between PFC activity and the affective-motivational dimension of approach/withdrawal (Davidson, 1992). Furthermore, approach has been shown to underpin higher-level emotions such as empathy (Light et al., 2009; Gilroy et al., 2013) and anger (Harmon-Jones, 2003).

In previous work, we have demonstrated that both anger and empathy could be been successfully used in a BCI context to interact with virtual agents (Gilroy et al., 2013; Aranyi et al., 2015b). Our objective here is to bridge the gap with affective expression and improve the joint analysis of objective and subjective users’ responses. Hence, as a component of rapport, approach is the positive element of input on the user’s side. PFC asymmetry can be measured through various signals including electroencephalography (EEG) (Davidson, 1992), functional magnetic resonance imaging (fMRI) (Zotev et al., 2013) and fNIRS (Doi et al., 2013). We have opted for fNIRS because it offers better signal stability and robustness to motion artifacts and also because PFC activity is readily accessible to fNIRS measurement, in particular the dorsolateral PFC (DL-PFC) through lateral optodes (Ayaz et al., 2011).

Following the definitions of *rapport* according to Gratch et al. (2007), in our setting the positive signal from the user towards the agent corresponds to the intensity of approach, and the positive response from the agent to the evolution of non-verbal behavior and facial expressions (combination of focus of attention and positively valenced expressions). The establishment of rapport being seen as the concomitance of positive signals on both sides in an interaction setting, it is thus well captured by the experimental design.

While previous studies have related the perception of rapport to PFC signals (Schilbach et al., 2006; Gordon et al., 2014), it is important to note that here, we are not directly equating rapport to PFC asymmetry. Instead, we show that rapport can emerge, from the user perspective, from the perception of a positively valenced response to her own engagement or mental disposition. We posit that a computational model of approach can support a visually realistic engagement with an agent displaying realistic affective behavior. This will be measured through the combination of successful NF in this interactive context with subjective experience questionnaires.

## Previous and Related Work

One of the earliest works using neuroimaging to study the perception of virtual agents is from Schilbach et al. (2006). They investigated neural correlates of observed social interactions with virtual agents using fMRI, the behaviors of virtual agents being designed to be perceived as either socially relevant or arbitrary. Their main finding was that perception of social interactions (defined as “adequate facial expressions” for the virtual agent) resulted in an activation of the ventromedial PFC (VM-PFC), which was not observed when the agents expressions were arbitrary. They attribute this activation to the VM-PFC’s involvement in the early processing of social meaning, also citing its role in joint attention as well as in guiding approach and withdrawal. In more recent work, they have explored an interactive setting for joint attention based on mutual gaze between the user and a virtual agent (Schilbach et al., 2010), showing reward-related neurocircuitry to be activated during the initiation of joint attention.

Nozawa and Kondo (2009) have monitored the activity of the PFC using fNIRS during controlled mouse-based interaction with a virtual agent and found a surge of activity predominantly in the DL-PFC, which they interpreted in attentional terms only. Benbasat et al. (2010) reported a neuroimaging study of how virtual agents induce social presence as a function of their appearance (gender, ethnicity) using fMRI, with the anterior paracingulate cortex as a region of interest.

Our own work, while clearly following on results from Schilbach et al. (2006, 2010), aims at deriving BCI techniques supporting social interaction with virtual agents rather than investigating the neural correlates of human-agent interaction. BCI input bypasses traditional expressive mechanisms of human subjects (facial expressions, gaze), while virtual agents use a range of facial expressions reflecting the user’s perceived level of engagement. We concentrate on the DL-PFC as a region of interest because of its relation to approach (Spielberg et al., 2012) and our previous experience with fNIRS-based NF (Aranyi et al., 2015a,b). While our installation is not fully ecological in terms of communication, by relying on users’ mental disposition it trades naturalness for controllability and sustained interaction.

Previous research in embodied conversational agents has investigated both empathic agents supporting the user in learning situations (Prendinger and Ishizuka, 2005) and agents that elicit empathy from users. The use of physiological signals has been experimented in both contexts (Prendinger and Ishizuka, 2005; Gilroy et al., 2013). Gilroy et al. (2013) have described the use of EEG prefrontal asymmetry to capture empathy towards a virtual agent during an interactive narrative, but have not explored the agent’s expressions; instead they used a simplified feedback mechanism based only on color saturation.

Agents display their emotional states through their verbal and non-verbal behaviors. While earlier models focused on the six prototypical expressions of emotion, latest models allow modeling a large variety of facial expressions as a combination of the expressions of emotions (Albrecht et al., 2005) as sequences of multimodal signals (Niewiadomski et al., 2011), or even as a blend of expressions (Niewiadomski and Pelachaud, 2007). Models may rely on pure combinatorial approaches where the expression of an emotion is the result of an algebraic combination of other expressions (Albrecht et al., 2005) of regions on the face (Bui et al., 2001; Niewiadomski and Pelachaud, 2007); other models have relied on corpora annotation (Niewiadomski et al., 2011) where videos are carefully annotated to extract multimodal signals expressing emotions. Others have applied a perceptual approach (Grammer and Oberzaucher, 2006; Etemad et al., 2015) where human users are asked to create expressions of the virtual agents for given emotions.

Huang et al. (2011) also report that virtual agents can create rapport during interactions with human users by generating proper verbal and non-verbal behaviors. This provides a setting whereby we assume that generated non-verbal behaviors (i.e., facial expressions) are the reflection of the human user, which thus creates a relationship between the user’s input signals and the resulting visual feedback generated by the virtual agent.

## System Overview

**Figure 1:**
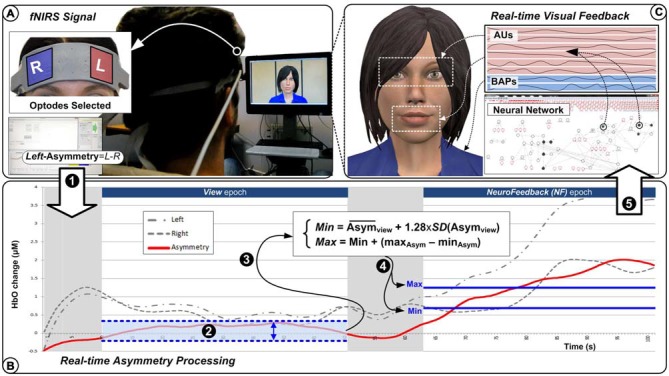
**System overview.** Brain signals are collected through functional near infrared spectroscopy (fNIRS) system **(A)** where left-most and right-most channels are processed to generate a left-asymmetry score (1). During the *View* epoch (2), the left-asymmetry values are used to define the *Min* and *Max* bounds (3) to be used during the *Neurofeedback (NF)* epoch where the real-time left-asymmetry scores (4) are normalized **(B)** before being used as single input (5) to the virtual agent’s facial expressions action units (AUs) and body action parameters under the neural network’s control **(C)**.

To support our experiments with virtual agent’s control from a single affective dimension, we designed a complete software platform, which is presented in Figure 1. From the user’s perspective, the virtual agent behaves autonomously as a response to what it perceives as the user’s mental disposition towards itself. Users are instructed to express positive feelings towards the agent in order to capture its interest. This should in turn result in the agent responding to the user with an expression matching the perceived interest in both valence and intensity.

Users’ attitudes are captured through their levels of PFC asymmetry, using fNIRS to measure differences in activity in left vs. right PFC (Figure 1A). This is based on extensive work relating the approach/withdrawal affective dimension to PFC asymmetry, measured through EEG (Davidson et al., 1990) as well as MRI (Zotev et al., 2014). We have adopted fNIRS because of its lesser sensitivity to artifacts compared to EEG and the fact that it is well-suited to capture activity in the PFC, in particular its dorsolateral region (Doi et al., 2013). Furthermore, experience gained when studying PFC asymmetry through fMRI (Cavazza et al., 2014) could provide guidance to design fNIRS experiments.

From the virtual agent’s perspective, the main challenge is to design an appropriate control process that would interpret and respond appropriately to the level of approach expressed mentally by the user. The system uses a network-like control representation to relate a single input to an array of action units (AUs) and body animation parameters (BAPs) that provide low-level control for the agent animation (Figure 1C). Our neural network behaves more like a graphical model with activation propagation than a traditional neural network with learning. It is designed to fine-tune control over a large number of variables with multiple control parameters (for further details, see Charles et al., 2015).

An overview of the operation of the system is as follows: the system captures PFC asymmetry in real-time and produces a matching virtual agent’s response. The PFC asymmetry baseline, which is subject-dependent, is acquired through an epoch during which subjects watch the virtual agent (displaying a neutral attitude) while carrying out a simple mental counting task (Figure 1B). During active engagement by subjects, PFC asymmetry is measured and its increase over the baseline is interpreted as the intensity of the approach towards the agent. Finally, the value is passed to the control network, which will generate matching virtual agent’s behavior, interrupting idle behavior and producing appropriate facial expressions.

## Virtual Agent

### Designing Agent’s Non-Verbal Behaviors

**Figure 2:**
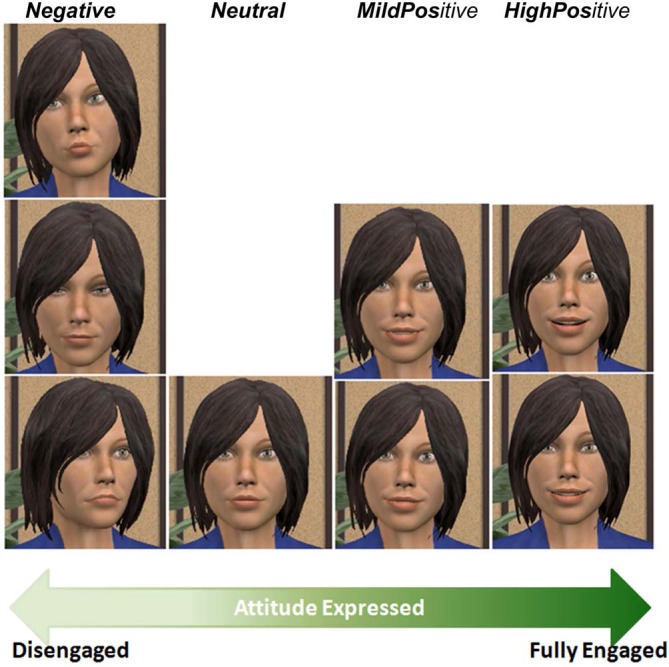
**Progression of the agent’s behaviors according to its level of engagement**.

To characterize the virtual agent’s range of non-verbal behaviors, we considered four levels of engagement, ranging from disengagement to full engagement. We designed non-verbal behaviors for our virtual agent corresponding to these four levels (see Figure 2). Gaze is one of the most prominent indicators of engagement. Indeed, gaze avoidance can be perceived as disengagement (Doherty-Sneddon et al., 2002), while mutual gaze is attributed to engagement (Burgoon et al., 1984). Moreover, empathic engagement can be achieved through affective interactions (Hall et al., 2005). Frequently expressing positively valenced emotions denote a high level of engagement, while negative expressions usually indicate disengagement.

The facial expressions of the virtual agent are defined in terms of AUs of facial action coding system (FACS) (Ekman et al., 2002). An expression is the combination of several AUs, where each AU corresponds to a muscular contraction. To design behaviors conveying an appropriate positive emotion, thus expressing engagement, we based our work on Ochs et al. (2016). Our combinations of AUs are slightly different than the ones described in Schilbach et al. (2006): we did not use winking (AU46), but added cheek rising (AU6) in combination with smiles, which is universally related to the expression of joy (Ekman et al., 2002). Moreover, we added jaw-dropping (AU26) to the mouth opening to model an expression of delight. The following AUs (AU1, AU2, AU5, AU12 and AU25) are used in both Shilbach’s work and ours. For body animations, our agent follows the MPEG-4 standard which defines body animation parameters (BAPs). Each BAP represents the rotation angle of one body joint along one axis.

Altogether, eight expressions were created for this experiment, following the literature (further details provided in Table 1):

- Low level of engagement: three behaviors depict a disengaged agent: (1, *Neg1*) The agent gazes away from the user, slightly tilts its head to the right, and shows a lip pout. (2, *Neg2*) The agent gazes completely down. (3, *Neg3*) The agent slightly turns its body away from the user and lowers its lips’ corners. The facial expressions generated used AU15, AU17, AU18, AU23 and AU24 for the mouth; and AU43, AU62 and AU64 for the eyes of the agent. Body movements generated used vc1roll, vc1tilt and vc1torsion for the cerebral vertebra and *vl1torsion* for the lumbar vertebra.
- Below average level of engagement: (4, *Neutral*) The agent shows a neutral facial expression with its gaze and body oriented towards the user.
- Above average level of engagement: two behaviors portray a mildly positive attitude, expressing happiness. (5, *MildPos1*) The agent keeps its gaze directed at the user and displays a smile of mild intensity. (6, *MildPos2*) The agent slightly tilts its head on the left, looks at the user, and displays a smile of mild intensity. The facial expressions generated used AU12, AU25 and AU26 for the mouth; and AU5 for the eyes of the agent. Body movement generated used *vc1roll* for the cerebral vertebra.
- High level of engagement: two behaviors match with the agent’s engagement as it displays delight. (7, *HighPos1*) Its smile widens, crow’s feet appear around the eyes as the cheeks are raised and its head slightly tilts backwards. (8, *HighPos2*) The agent’s smile widens, it slightly opens its mouth and raises its eyebrows. The facial expressions generated used AU1 and AU2 for the eyebrows; AU6 for the cheeks; AU12, AU25 and AU26 for the mouth; and AU5 for the eyes of the agent. Body movement generated used vc1tilt for the cerebral vertebra.

**Table 1:** **Details of the action units (AUs) and body movement defined to encode the facial expressions and behaviors for the each level of engagement**.

		Engagement level			
		Negative	Neutral	Mild positive	High positive
AU family	Eyebrows				AUl (Inner brow raiser)
					AU2 (Outer brow raiser)
	Eyes	AU43 (Eyes closed)		AU5 (Upper lid raiser)	AU5 (Upper lid raiser)
		AU62 (Eyes right)			
		AU64 (Eyes down)			
		AU61 (Eyes left)			
	Cheeks				AU6 (Cheek raiser)
	Mouth	AU23 (Lip tightener)		AU12 (Lip corner puller)	AU12 (Lip corner puller)
		AU24 (Lip pressor)		AU25 (Lips part)	AU25 (Lips part)
		AU18 (Lip pucker)		AU26 (Jaw drop)	AU26 (Jaw drop)
		AU15 (Lip corner depressor)			
		AU17 (Chin raiser)			
BAP		vc1roll (Cervical vertebra roll)		vc1roll (Cervical vertebra roll)	vc1tilt (Cervical vertebra tilt)
		vc1tiIt (Cervical vertebra tilt)
		vc1torsion (Cervical vertebra torsion)
		vl1torsion (Lumbar vertebra torsion)

### Designing Natural Transitions Between Behaviors

To ensure the provision of continuous natural feedback to the user, the different behaviors are mapped onto one other. We developed transitions between behaviors representing different levels of engagement. Each behavior corresponds to a particular combination of AUs and BAPs. To avoid uncanny animations (e.g., when the agent’s expression freezes) and to obtain smooth transitions between two behaviors, we designed an interpolation function (based on Huang and Pelachaud, 2012) so that the previous behavior fades away while the new one slowly appears, resulting in a blended behavior.

Having different behaviors for the same level of engagement avoids the uncanny repetition of similar expressions and increases realism. The variation of behaviors also ensures that the agent does not freeze when it remains at the same level for a long time. Hence, the agent displays a new behavior whenever the user maintains a particular level of left-asymmetry for around 2 s.

To validate the behaviors displayed by the agent, therefore to ensure giving an adequate feedback to the user, we conducted two evaluation studies. In the first one, we asked participants to evaluate each of the agent’s non-verbal behaviors according to their valence (negative or positive) and their naturalness. In the second one, we asked participants to rank four different behaviors using the same valence scale, from the least positive to the most positive.

### Evaluating the Perceived Valence of Non-Verbal Behaviors

The purpose of our evaluation was to test the following hypothesis (H1): the valence of the agents’ non-verbal behaviors is appropriately perceived.

We generated eight videos. In each video, we captured the transition from the neutral behavior to one of the eight non-verbal behaviors we designed (see Figure 2). After the transition, the agent maintained its behavior for almost 2 s. All the videos were generated with the same procedure: same appearance of the virtual agent, same position of the camera, same duration of the animation (approximately 4 s). All the videos last exactly 2.3 s and were made the same way: the agent keeps a neutral expression for 0.15 s, then expresses a non-verbal behavior for 2 s and finally maintains a neutral expression for 0.15 s. There was no sound in the videos. We asked subjects to watch each of the eight videos through a web study. After watching each video, subjects rated the valence of the feeling expressed by the agent using 7-point labeled Likert scales. To avoid carryover effects during the evaluation, we used a Latin square design to generate the order of the videos. We recruited a total of 16 participants via mailing lists (56% male). Most of the subjects were French (75%), and between 25 and 35 years old (82%).

**Figure 3:**
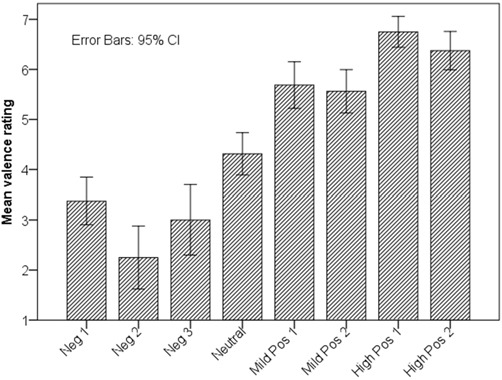
**Valence ratings associated with AU and BAP combinations.** Note that the four facial-expression categories (negative, neutral, mildly positive, and highly positive) were rated in the intended order.

In order to test H1, we ran a one-way repeated measures analysis of variance (ANOVA) with perceived valence as dependent variable. The within-subjects factor was AU and BAP combinations (with eight levels; Figure 2). There was a statistically significant effect of AU and BAP combinations on perceived valence, *F*_(7,105)_ = 53.91, *p* < 0.001, *η*^2^ is 0.78 (large). Figure 3 shows average valence ratings for each AU and BAP combination with 95% confidence interval (CI). To assess whether the four categories of agent behavior (negative, neutral, mildly positive, and highly positive) were perceived reliably differently, we performed three *post hoc* pairwise comparisons using Bonferroni correction (one-tailed *p* < 0.05 criterion adjusted for three comparisons: *p* < 0.017): (1) highest-rated negative vs. neutral; (2) neutral vs. lowest-rated mild positive; and (3) highest-rated mild positive vs. lowest-rated high positive. Because the parametric assumption of normality was violated for half of the AU and BAP combinations, we report pairwise related-samples comparisons using Wilcoxon Signed Ranks Test.

Comparison 1: subjects rated the valence of neutral agent expression (Neutral; *M* = 4.31, SD = 0.79) significantly higher than that of the highest-rated negative agent expression (Neg1; *M* = 3.38, SD = 0.89), *T* = 12, *z* = −2.66, *p* (one-tailed) = 0.004, *r* = 0.66 (large). Comparison 2: subjects rated the valence of the lowest-rated mild positive agent expression (MildPos2: *M* = 5.56, SD = 0.81) significantly higher than the neutral agent expression, *T* = 11, *z* = −3.04, *p* (one-tailed) = 0.001, *r* = 0.76 (large). Comparison 3: subjects rated the valence of the lowest-rated high positive agent expression (HighPos 2; *M* = 6.38, SD = 0.72) significantly higher than that of the highest-rated mild positive agent expression (MildPos1; *M* = 5.69, SD = 0.87), *T* = 3, *z* = −2.37, *p* (one-tailed) = 0.009, *r* = 0.59 (large).

H1 was supported: users could differentiate between the different facial expressions of the agent’s non-verbal behaviors, the valence ratings were in line with the intended order, and the differences between the four main categories of the agent’s facial expressions were characterized with a large effect size. To further investigate if the non-verbal behavior of the agent is interpreted as intended, we conducted a second evaluation addressing the following hypothesis (H2): the non-verbal behaviors validated in the previous evaluation are correctly ranked according to their valence.

We kept the same videos as for our first evaluation and we designed a drag and drop interface allowing users to rank the behaviors expressed in the videos according to perceived valence. To avoid carryover effects during the evaluation, we used a Latin square design to generate the order of the combinations. We recruited a total of 36 subjects via mailing lists (61% male). Most of the subjects were French (61%), and between 25 and 35 years old (81%).

We coded the ranking of the behaviors into numerical valence values and computed the mean valence score of each behavior. The results confirmed the ones obtained during the first evaluation: the eight behaviors could be clustered into different groups according to their valence. We checked whether each of the eight behaviors was ranked according to our previous findings. Most of them were correctly ranked (*M* ≥ 90%) except for one negative behavior (Neg1: *M* = 67.36) and the neutral behavior (Neutral: *M* = 85.88). The behavior Neg1 representing a pouting behavior was sometimes considered more positive than the neutral behavior. This can be explained as the pouting AU is not directly related with negatively valenced emotions, unlike the AU used for the behaviors Neg2 and Neg3. We can also hypothesize that Pouting expressions may be found culturally acceptable rather than negative, especially considering the subjects’ age range. Overall, the results supported H2.

## Materials and Methods

### BCI Input

We used an fNIR400 Optical Brain Imaging Station by Biopac Systems, with a 16-channel sensor and a fixed 2.5 cm source-detector separation for BCI input (for channel locations, see Ruocco et al., 2014). This device measures intensity changes in two wavelengths (730 nm and 850 nm) to calculate changes in oxygenated and deoxygenated hemoglobin concentration (HbO and HbR, respectively) using the modified Beer-Lambert law (see Ayaz, 2010). Raw data and HbO values were collected with 2 Hz sampling frequency using software provided by the device manufacturer (COBI Studio and FnirSoft v4.1), and was sent to a remote client experimental software over TCP/IP (using FnirSoft DAQ Tools).

We selected the HbO signal to provide real-time feedback, which has been applied successfully in research involving affect-related manipulation in the DL-PFC (Tuscan et al., 2013), and in the context of approach/withdrawal-related experimental manipulation (Morinaga et al., 2007). Furthermore, HbO has been reported to be more sensitive to changes in cerebral blood flow than HbR and total (HbT) hemoglobin changes (Hoshi, 2003), and it is characterized with a higher signal-to-noise ration then HbR and HbT (Strangman et al., 2002). Since we used HbO for real-time mapping to the feedback channel, we report *post hoc* analyses based on the same signal for consistency.

We operationalized BCI input based on asymmetric functional activation in the DL-PFC. A metric of asymmetry was derived real-time by the experimental software by averaging HbO values across the four channels located over the left and right DL-PFC, respectively, then subtracting average Right from average Left. This value, updated with the same 2 Hz frequency of the sampling rate, reflects inter-hemispheric difference in HbO change in micromolar units (μMol/L).

**Figure 4:**
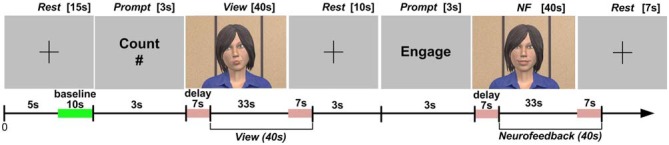
**Protocol design, including setting the baseline of the fNIRS system, as well as windowing the data collection during the *View* and *NF* epochs to account for the delay in hemodynamic response**.

The collection of fNIRS data was organized into blocks, each consisting of a sequence of epochs (short time periods with a specific task). We describe the structure of blocks and the experimental tasks during each epoch in the next section. HbO values on each channel were extracted using time synchronization markers. To compensate for the approximately 7 s delay in the hemodynamic response (Bunce et al., 2006), we excluded data from the first 7 s in each epoch and included data from the first 7 s after the completion of each epoch (Figure 4; Aranyi et al., 2015a).

### Subjects, Procedure, and Feedback Mapping

Eighteen english-speaking adult subjects participated in the experiment (eight female, mean age = 35.11 years, SD = 11.25, range: [21; 60]). Data from one subject was discarded due to technical problems during data collection. Subjects were right-handed and were not treated for psychiatric conditions. The study was approved by Teesside University’s Research Ethics Committee. Subjects provided written consent prior to participation and received an online retailer voucher equivalent to $30 upon completing the experimental protocol.

We followed the recommendations of Solovey et al. (2009) for the use of fNIRS in a HCI setting. Subjects seated approximately 47″ (120 cm) away from a 24″ monitor displaying experimental instructions and stimuli (including visual feedback during NF) in a dimly-lit, quiet room in a comfortable chair to minimize movement artifacts, with the fNIRS sensor positioned on their forehead, covered with non-transparent material to prevent ambient light from reaching the sensors. Subjects were instructed to refrain from talking, frowning and moving their limbs during fNIRS data collection periods within the protocol. Additionally, we applied sliding-window motion artifact rejection (SMAR; Ayaz et al., 2010); each channel used for calculating the asymmetry metric was inspected *post hoc* to identify motion artifacts during NF. For *post hoc* analyses, raw data were low-pass filtered using a finite impulse response filter with order 20 and 0.1 Hz cut-off frequency (Ayaz et al., 2010).

Following instructions and a practice block, each participant completed eight identical blocks, consisting of three epochs: *Rest*, *View*, and *NF*. Block design and the timing of epochs are presented in Figure 4 (also see Charles et al., 2015).

During *Rest*, subjects were instructed to look at a crosshair at the center of a gray screen, to try to clear their head of thoughts and relax. HbO for each channel within a block was calculated with respect to a baseline measured during the last 10 s of this *Rest* epoch. During *View*, subjects were instructed to look at the virtual agent while carrying out a mental arithmetic task: counting backwards from 500 by increments of an integer provided before the epoch in a visual prompt lasting 3 s. Backward counting is the most commonly used mental arithmetic task in fNIRS research (Naseer and Hong, 2015); it was included to control for unwanted mental processes while looking at the virtual agent (see Sarkheil et al., 2015). Subjects were informed that the agent would be unresponsive to their brain input during *View*.

During *NF*, subjects were instructed to attempt to cheer up the virtual agent using their thoughts. *NF* was separated from *View* by a 10 s *Rest* epoch, and preceded by a 3 s prompt to start engaging with the virtual agent through (positive) thinking. Subjects were told that the agent would be responsive to their brain input during *NF*, and to expect a few seconds delay in the agent’s reactions due to the nature of the brain input used in the study. Since, due to this delay, the effectiveness of any cognitive strategy could not be seen immediately, we also asked subjects to be patient and apply a strategy consistently throughout a block. They were also shown how a happy agent would look like (i.e., the target state) before the practice block preceding data collection. We were deliberately vague with instructions regarding how to cheer up the agent in order to allow subjects to develop their own strategies. *NF* was followed by a final *Rest* epoch lasting 7 s. After completing each block, subjects were asked to describe the strategy they used during *NF* in broad terms. Additionally, they rated the extent to which the virtual agent’s facial expressions were appropriate to their thought contents during *NF* on a 1–7 scale. This rating was included to estimate the subjective level of alignment between the subject’s thoughts and the agent’s expressive behavior in each block.

**Figure 5:**
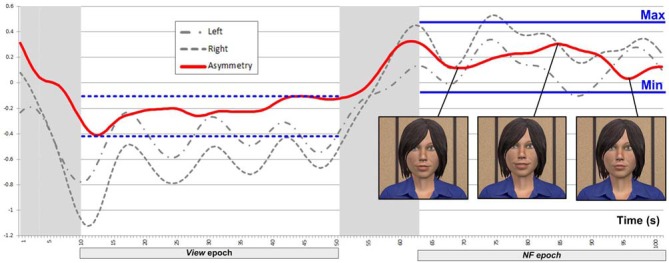
**Example of a successful block, where asymmetry during *NF* is significantly larger than during *View***.

We included the *View* epoch in each block as a reference (with the mental arithmetic task unrelated to asymmetry) to support feedback mapping for *NF* within the same block, in the following way. We defined the threshold (i.e., minimum asymmetry value resulting in feedback) during *NF* based on asymmetry values collected during *View* within the same block (Figure 5). The minimum point (Min) for mapping was defined as the mean of asymmetry values during the *View* epoch plus 1.28 times their SD. Assuming normally distributed asymmetry scores, this threshold would result in no feedback for 90% of the asymmetry values during *View*. This approach to determine NF threshold is consistent with the original one of Rosenfeld et al. (1995) for EEG-based frontal-asymmetry NF. To determine the maximum point (Max) for mapping (i.e., the asymmetry value resulting in feedback with maximum magnitude), we added the variation range of asymmetry values during *View* to the threshold Min we defined above. Outliers (values outside three SDs from the mean) were removed for calculating the threshold and range in order to prevent extreme values, likely resulting from movement artifacts, exerting an unduly influence on NF mapping. Asymmetry values within the range [Min; Max] during *NF* were mapped linearly onto the virtual agent’s facial expression, with the same 2 Hz frequency as the acquisition of asymmetry values.

Note that *View* and *NF* epochs were matched for length and for visual stimulus, with the exception that during *NF*, subjects could influence the agent’s expressions. Since subjects’ mental activity was controlled during *View* with a mental arithmetic task theoretically unrelated to DL-PFC asymmetry, increase in asymmetry can be attributed to changes mental activity between the *View* and *NF* epochs. This way, using a reference epoch provides a control condition within each block. Rather than providing absolute levels of HbO and HbR, the collected values reflect changes relative to a baseline (Ayaz, 2010), they can be difficult to compare across subjects (Sakatani et al., 2013), and the magnitude of HbO changes can differ substantially across blocks within the same subjects. However, the above strategy for determining feedback threshold and range on a per-block basis mitigates these issues and promotes the comparability of *NF* epochs both within and between subjects.

## Results and Discussion

After data screening and filtering, we identified 136 valid blocks for analysis (eight blocks were collected from each 17 subjects). We defined block success as a statistically significant increase in average asymmetry during *NF* compared to the *View* epoch within the same block. Block success was determined *post hoc* using independent *t*-test with bootstrapping resampling method (1000 samples, 95% CI) with epoch type (View/NF) as independent variable and asymmetry scores as dependent variable, applying Bonferroni correction on a subject basis to control false discovery rate (one-tailed *p* threshold of 0.0125). (Note that the entire NF epoch was considered to determine block success, not just above-threshold asymmetry values.) Based on this criterion, 70 out of the total 136 blocks (51%) were successful. For a detailed discussion of the design of experimental blocks to support the use of this success criterion, see Aranyi et al. (2015a).

**Figure 6:**
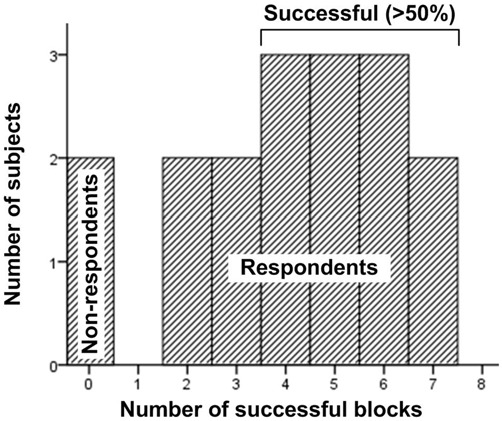
**The distribution of block success across subjects**.

Based on their NF performance, we classified subjects as respondents (completed at least one block successfully) and non-respondents (completed no successful blocks). Fifteen of the seventeen valid subjects (88%) were classified as respondents. Additionally, the two non-respondents were not simply failed on the statistical criterion; they have not crossed the feedback threshold at all during the experiment (i.e., received no feedback whatsoever). Eleven out of the 15 respondents (73%) completed at least half of the blocks successfully (Median = 4). No subject completed all blocks successfully (Figure 6). This success rate on the subject basis is identical to what we observed in a separate study applying the same NF protocol (Charles et al., 2015).

The dichotomous (NF success/fail) statistical success criterion described above does not contain information about the magnitude of asymmetry up-regulation during successful NF. Additionally, the experimental protocol was designed considering the fixed 2 Hz sampling rate of the fNIRS system we used to achieve sufficient statistical power to detect changes in asymmetry. Therefore, we also calculated effect-size metrics (*r* and Cohen’s *d*) to characterize the magnitude of NF success, rather than solely relying on statistical significance depending on sampling frequency.

**Figure 7:**
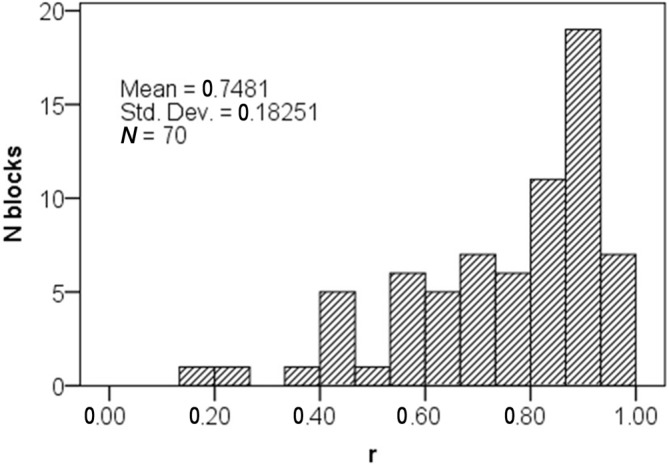
**Distribution of the effect-size measure *r* in successful blocks**.

The effect-size *r* is interpreted similarly to the correlation coefficient, its value is constrained between 0 and 1, and according to Cohen (1988) conventions, *r* = 0.10 corresponds to small, 0.30 to medium, and 0.50 to large effect-size. The smallest *r* we could detect was 0.18 (small), and average *r* in successful blocks was 0.75 (large). Furthermore, the distribution of *r* in successful blocks was negatively skewed (Median *r* = 0.81), indicating that successful blocks were predominantly characterized with large asymmetry increase (Figure 7).

We calculated Cohen’s *d* by dividing the difference between mean asymmetry during the *NF* and *View* epochs with the pooled SD of asymmetry scores. The resulting *d* value reflects the distance between the distributions of asymmetry scores collected during successive reference and *NF* epochs, which can be readily interpreted. Mean *d* in successful blocks was 2.58 (*SD* = 1.41), which corresponds to an average 20% overlap in asymmetry scores between successive *View* and *NF* epochs (assuming normally distributed values), with a 96% chance that an asymmetry value picked randomly from the *NF* epoch will be greater than a random asymmetry value from the *View* epoch (probability of superiority). The smallest effect we could detect (with 40 s epochs sampled at 2 Hz) was *d* = 0.35 (small), which corresponds to 86% overlap and 60% probability of superiority. This magnitude of increase in asymmetry is comparable to our previous study, where subjects up-regulated left-asymmetry by mentally expressing anger (a negatively valenced, but approach-related affect) towards a virtual agent who was previously identified as mischievous in a narrative context (see Aranyi et al., 2015b).

These findings indicate that our success criterion was sensitive to detect small increases in asymmetry, while asymmetry values on average had small overlap between *View* and *NF* epochs: successful blocks were characterized with a marked increase in asymmetry during *NF*. Additionally, in successful blocks, the *r* and *d* effect-size measures were positively and significantly correlated with perceived alignment (the subjective rating of how appropriate were the virtual agent’s facial expressions to the subject’s thoughts during NF): *r* = 0.56, and *r* = 0.45, respectively (both* p* < 0.001). This provides validation from self-report data for the use of effect-size metrics to characterize the magnitude of NF success.

Since our experiment consisted of a single session, no training effect was expected. However, a possible practice effect would consist in a linear increase in the proportion of Block Success across subjects with time (from Block 1 to 8). Since Block Success was a continuous dichotomy, we calculated the biserial correlation coefficient to assess the linear relationship between Block Success (Yes/No) and block number (1–8). The analysis found no practice effect, *r_b_* = 0.03, *p* = 0.76, *ns*.

**Figure 8:**
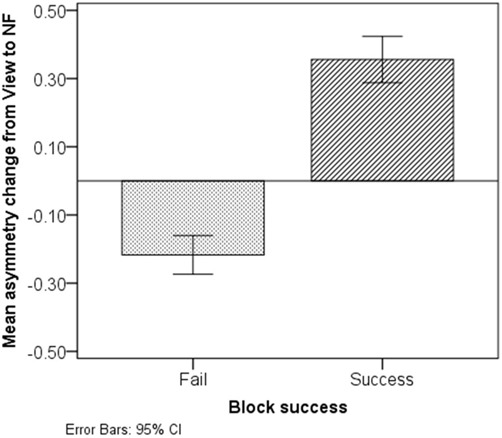
**Asymmetry change from *View* to *NF* in non-successful and successful blocks.** Successful blocks are characterized with a marked asymmetry increase during *NF*, while non-successful ones are characterized with a slight decrease.

Figure 8 presents average asymmetry change between successive *View* and *NF* epochs. Successful blocks were characterized with a marked increase in asymmetry during *NF* (*M* = 0.36, SD = 0.29); conversely, non-successful blocks were characterized with a comparably small asymmetry decrease during NF (*M* = −0.22, SD = 0.23).

We explored how HbO changes on the left and right sides contributed to asymmetry increase from *View* to *NF* in successful blocks by conducting a two-way within-subject ANOVA with average HbO change as dependent variable, and Epoch type (*View/NF*) and Side (*Left/Right*) as factors.

**Figure 9:**
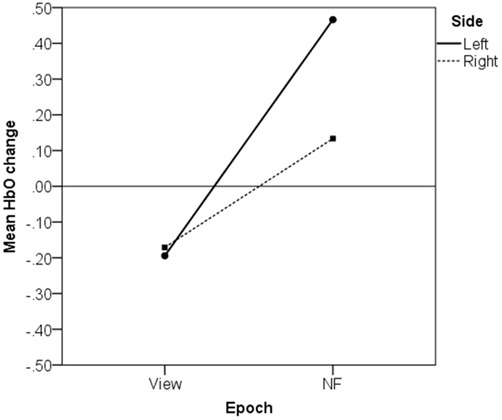
**Left-lateralized increase in average oxygenated hemoglobin concentration (HbO) in successful blocks**.

The analysis revealed a significant interaction, *F*_(1,69)_ = 108.76, *p* < 0.001, partial *η^2^* = 0.61 (large), which indicates that HbO changed differentially on the left and right sides between consecutive *View* and *NF* epochs. This interaction is displayed in Figure 9, showing that average HbO change during *View* (with counting reference task) did not display lateralized difference, *t*_(69)_ = 0.53, *p* = 0.595, n.s., *r* = 0.06 (small), while average HbO increase on the left size (*M* = 0.47, SD = 1.05) was significantly and substantially larger than on the right (*M* = 0.13, SD = 1.07), *t*_(69)_ = 6.73, *p* < 0.001, *r* = 0.63 (large).

Conversely, non-successful blocks were characterized by stability, or even a decrease in left asymmetry (Figure 8). Out of the 66 failed blocks, 15 (23%) showed no statistically significant change in asymmetry from *View* to *NF*, while 51 (77%) exhibited a significant decrease in left-asymmetry. Replicating the above two-way within-subject ANOVA on non-successful blocks revealed a significant interaction of Epoch type (*View/NF*) and Side (*Left/Right*), *F*_(1,65)_ = 58.11, *p* < 0.001, partial *η^2^* = 0.47 (large). In failed blocks, average HbO during *View* did not display lateralized difference, *t*_(65)_ = −0.33, *p* = 0.740, *ns*, *r* = 0.04 (small), while during *NF*, average HbO change on the left size (*M* = 0.24, *SD* = 0.98) was significantly lower than on the right (*M* = 0.47, *SD* = 0.91), *t*_(65)_ = −3.93, *p* < 0.001, *r* = 0.44 (medium). This is not consistent with our previous findings using a similar protocol (Aranyi et al., 2015b), where we found no lateralized changes in HbO in failed blocks.

These findings show that 51 out of the 136 blocks (37.5%) collected in the experiment where characterized with a decrease in left-asymmetry while subjects reported using cognitive strategies traditionally associated with left-asymmetry up-regulation. However, we note that 13 (25%) of these blocks were actually produced by the two non-responsive subjects (i.e., those who had not completed a single successful block). It also needs to be noted that our subjects did not receive NF training spread over multiple sessions prior to the experiment (e.g., see Rosenfeld et al., 1995; Kotchoubey et al., 2002). Here, subjects’ interaction with the system was restricted to a single short session (eight blocks including a 40 s NF epoch each, plus a practice block); therefore, no learning effects could be expected, while practice effects within a single session were confounded with the effect of fatigue (ratings of task-difficulty are discussed below).

**Figure 10:**
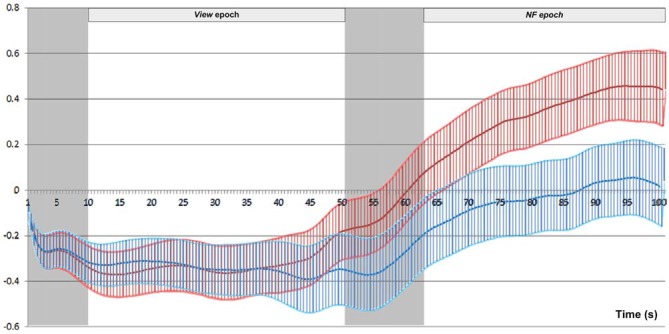
**Mean and standard error of HbO across successful blocks (*N* = 70), for left (red) and right (blue) sides separately.** The signal on the two sides overlaps completely during *View*, while left rises above right during *NF*.

**Figure 11:**
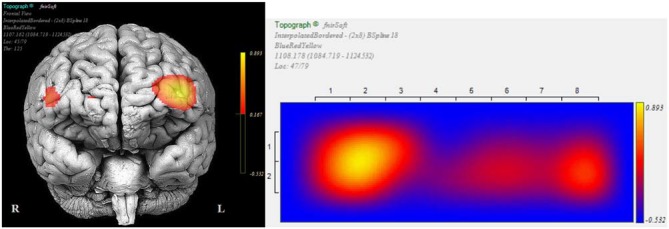
**Topographic snapshots of asymmetric HbO increase in the dorsolateral prefrontal cortex (DL-PFC) during a successful *NF* epoch**.

We conducted *post hoc* pairwise comparisons to breakdown the significant interaction effect of Epoch type (*View/NF*) and Side (*Left/Right*) in successful blocks. On the left side, average HbO increase from *View* (*M* = −0.19, SD = 0.80) to *NF* (*M* = 0.47, SD = 1.05) was statistically significant, *t*_(69)_ = 9.44, *p* < 0.001, *r* = 0.75 (large). On the right side, average HbO increase from *View* (*M* = −0.17, SD = 0.70) to *NF* (*M* = 0.13, SD = 1.07) was also statistically significant, but with a markedly lower effect-size, *t*_(69)_ = 3.80, *p* < 0.001, *r* = 0.42 (medium). These findings indicated that although mean HbO increased on both sides during successful *NF*, asymmetry resulted from a more pronounced HbO increase on the left side (Figure 10 illustrates for across successful blocks, whilst Figure 11 shows an example of a single successful *NF* epoch).

This bilateral increase in HbO may result from increased mental effort when switching from the counting task to NF. Subjects rated the subjective difficulty of both tasks on a 1–7 Likert scale after completing the protocol. Wilcoxon signed-rank test revealed that subjects rated the *NF* task (*M* = 5.18, SD = 1.13) more difficult than the *Count* task (*M* = 4.12, SD = 1.50), *T* = 20, *z* = 2.321, *p* = 0.020, *r* = 0.56 (large). Additionally, each subject answered six questions extracted from a generic rapport questionnaire (Gratch et al., 2007), rated on a 1–7 Likert scale, after completing the experimental protocol. The scale had satisfactory internal consistency (Cronbach’s alpha = 0.73). We calculated Spearman’s rho non-parametric correlation coefficient to explore the relationship between alignment and NF success. There was a strong and statistically significant positive relationship between perceived alignment the number of successfully completed blocks, *r_s_* = 0.62, *p* < 0.01.

Following each block, subjects were asked to describe their cognitive strategies to engage with the virtual agent, and thought contents during NF epochs (regardless of the amount of feedback they received during the *NF* epoch). We identified two broad categories of cognitive strategy (i.e., a set of thought contents to concentrate on during *NF*): direct and indirect. Direct strategies involved trying to cheer up the agent in an internal dialog, imagery involving the agent (e.g., reaching out to her), expressing empathy or otherwise engaging mentally with her. Indirect strategies involved recollection of pleasant personal memories, imagery of pleasant activity unrelated to the agent, and thinking about pleasant things (e.g., singing a song in one’s head). Chi-squared test revealed no significant association between blocks success (*Success/Fail*) and strategy type (*Direct/Indirect*), χ^2^_(1)_ = 2.52, *p* = 0.112, Cramer’s *V* = 0.14 (small). Indirect strategy is more prominent in failed blocks (41 out of 66; 62%) whilst, in successful blocks, there was an almost even split in the use of direct and indirect strategies (36 and 34, respectively).

## Conclusion

Our results confirm that users are able to engage with virtual agents by controlling their mental disposition, through thought contents and cognitive strategies. However, this is the first complete report of a context which is behaviorally realistic on the agent side (unlike, for instance, (Gilroy et al., 2013), which used color saturation instead of realistic facial expressions), and in which BCI input is analyzed together with user experience subjective data.

Previous work has associated rapport primarily with activation of the VM-PFC (Gupta Gordon et al., 2014), while empathy has been considered closely related to approach, whose relevant area is the DL-PFC. At the same time, VM-PFC is generally considered inaccessible to fNIRS (Doi et al., 2013), and our results have evidenced a significant correlation between self-reported magnitude of rapport (characterized as perceived alignment) and DL-PFC asymmetry. To resolve this apparent contradiction, we should note that the definition of rapport has been sometimes strongly influenced by alignment (Menenti et al., 2012) and mimicry (Lakin and Chartrand, 2003; Wang and Hamilton, 2015), in which the affective component is less prominent. Our definition of rapport is closer to empathy without identifying entirely with it, because of the lack of background information on the virtual agent and the fact that it is not expressing a pre-existing emotional state.

A potential limitation of these experiments is the non-dissociation between approach and valence when measuring PFC asymmetry, valence also impacting activity in the DL-PFC (Berkman and Lieberman, 2010). This can be also evidenced by the subjects’ self-reporting of cognitive strategies, which showed an even split between cognitive strategies emphasizing approach (reaching out, targeting the agent itself), and those emphasizing positive thoughts in general [often self-centered, as previously reported in other, fMRI-based PFC asymmetry NF experiments (Zotev et al., 2014)]. However, in terms of user experience, the agent’s response was perceived as appropriate regardless of the actual cognitive strategy employed. Strictly speaking, the true “rapport” hypothesis is only fully realized when the subject’s cognitive strategies targets the agent itself, the issue with positive thoughts strategies not being so much the use of valence but the fact that some positive thoughts could be self-centered. The above finding is attributable to individual subjects adopting conservative cognitive strategies, rather than to an inherent limitation of the model: this can be explained in particular considering the limited NF training that subjects have been offered. In earlier work, we have demonstrated that successful interaction with virtual agents could be realized through approach alone, by using an anger paradigm (Aranyi et al., 2015b), in which approach is fully dissociated from valence (Harmon-Jones, 2003). In the context of rapport, a mixed contribution from approach and valence seems unavoidable, the decisive factor being that positive thoughts should be directed at the agent rather than self-centered.

## Funding

This work was supported by the European project H2020 ARIA-VALUSPA (No 645378) and the French ANR project MOCA (ANR-12-CORD-019). It was partially performed within the Labex SMART (ANR-11-LABX-65) supported by French state funds managed by the ANR within the Investissements d’Avenir programme under reference ANR-11-IDEX-0004-02.

## Author Contributions

GA: (BCI) protocol design, experimental setup, experiments, statistical analysis, write-up. FP: (ECA) software design and implementation, ECA experimental setup and experiments, write-up. FC: (BCI) software design and implementation, experiments, write-up. CP: development of research question, research expertise and guidance, write-up. MC: development of research question, research expertise and guidance, write-up.

## Conflict of Interest Statement

The authors declare that the research was conducted in the absence of any commercial or financial relationships that could be construed as a potential conflict of interest.
